# Perampanel and Visuospatial Skills in Children With Epilepsy

**DOI:** 10.3389/fneur.2021.696946

**Published:** 2021-07-08

**Authors:** Francesca Felicia Operto, Valentina Vivenzio, Chiara Scuoppo, Chiara Padovano, Michele Roccella, Giuseppe Quatrosi, Grazia Maria Giovanna Pastorino

**Affiliations:** ^1^Child and Adolescent Neuropsychiatry Unit, Department of Medicine, Surgery and Dentistry, University of Salerno, Salerno, Italy; ^2^Department of Health Promotion, Mother and Child Care, Internal Medicine and Medical Specialties, University of Palermo, Palermo, Italy

**Keywords:** perampanel, visuospatial memory, tolerability, adverse effects, children

## Abstract

**Introduction:** Perampanel (PER) is a non-competitive AMPA glutamate receptor antagonist approved for focal and generalized seizures as add-on therapy. PER does not seem to negatively affect the cognitive profile in children and adolescents, but its influence on visuospatial abilities is still to be assessed. The aim of our study was to assess visuospatial skills through a standardized neuropsychological evaluation in adolescents taking PER for 12 months.

**Methods:** Our sample included 46 adolescents aged 12–18 years with focal and generalized drug-resistant epilepsy already in therapy with one or two antiseizure medications. Changes in visuospatial perception and memory were assessed by the Rey–Osterrieth Complex Figure Test at baseline (before taking PER) and after 12 months of pharmacological treatment. Executive functions and non-verbal intelligence were also assessed at baseline.

**Results:** After 12 months of PER therapy, the mean scores on the Rey–Osterrieth Complex Figure Test remained almost unchanged for both visuospatial perception and visuospatial memory skills. At baseline, visuospatial memory was related to executive function, and visuospatial perception was related to executive function and non-verbal intelligence.

**Conclusions:** Adjunctive treatment with PER did not negatively affect visuospatial skills. No adverse event effects have been reported after 12 months of follow-up, and this suggests a good tolerability in the middle-to-long term.

## Introduction

Perampanel (PER) is a relatively new antiseizure medication (ASM), approved as an additional treatment for focal-onset seizures with or without loss of awareness and primary generalized seizures in epileptic patients aged 4 years and older (1–3). Its peculiarity consists of a novel mechanism of action, different from other ASMs: PER is a selective, non-competitive antagonist to the α-amino-3-hydroxy-5-methyl-4-isoxazolepropionic acid (AMPA) receptor, an ionotropic glutamate receptor, that plays a basic part in fast excitatory synaptic transmission (4). Three phase III randomized controlled studies and subsequent open-label extension studies demonstrate the efficacy of PER as an add-on treatment of refractory focal seizures in adolescents (5–9). A subsequent multicenter, randomized, placebo-controlled study showed its efficacy in primary generalized tonic-clonic seizures in 164 patients aged 12 years and older (10).

More recently, other studies also highlighted good efficacy of PER in children from 4 years of age with different types of drug-resistant seizures (11, 12). Studies in the literature on the safety and efficacy of PER in children and adolescents have shown an overall favor risk–benefit profile with generally mild or moderate adverse effects (AEs). The most common AEs reported were dizziness (20.4%), drowsiness (15.3%), aggression (8.2%), decreased appetite (6.1%), and rhinitis (5.1%). It appears that the incidence of AEs is lower with a slower titration rate and that the AEs are reversible with the discontinuation of PER or with a dose adjustment (13).

In more recent years, tolerability research has paid increasing attention to the AEs of ASMs on cognitive function, especially in children and adolescents, whose central nervous system is still developing (14, 15). Regarding PER, the data currently available seem encouraging. A recent randomized, placebo-controlled study in adolescents with epilepsy revealed that PER had no negative effects on the global cognitive profile (16). Furthermore, executive functions, such as attention and working memory, also did not appear to be negatively affected by add-on therapy with PER in adolescent patients (17).

With the present study, we tried to fill a gap in the literature, by focusing on visual–spatial cognitive abilities and their changes during PER therapy. These abilities are important in various of contexts and human actions, such as movement, non-verbal communication, spatial orientation, spatial representation, graphic production, geometric recognition, and in disparate types of problem solving and academic achievements (18, 19).

Therefore, the primary aim of our prospective observational study was to evaluate visual–spatial skills through the use of standardized tests in adolescents with focal and generalized drug-resistant epilepsy at baseline and after 12 months of add-on treatment with PER.

A secondary objective of our study was to correlate visuospatial skills with executive functions, non-verbal intelligence and some demographics and epilepsy-related characteristics (age, sex, epilepsy duration, age at onset of epilepsy, frequency of seizure, seizure type, ASM number, PER dose).

## Materials and Methods

### Study Design and Participants

Our work is a prospective observational study. The patients were consecutively recruited at the Child Neuropsychiatry Unit of the University of Salerno from January 2016 to January 2020.

The eligibility criteria were as follows: focal or generalized epilepsy not well-controlled by drug therapy with one or two anticonvulsant drugs, non-verbal IQ above the fifth percentile [intelligence quotient (IQ) ≥ 70] evaluated with Raven's progressive matrices, and normal or corrected vision. Exclusion criteria were additional neurological (cerebral palsy, neurodegenerative diseases, or migraine), psychiatric (intellectual disability, attention deficit/hyperactivity disorder, specific learning disorder, anxiety, depression, and psychosis), or other relevant medical conditions (endocrinopathies, metabolic, hepatic, cardiac, or renal disorders) that could negatively affect neuropsychological performances.

Two different epilepsy clinicians made the diagnosis using clinical observations and electroencephalogram (EEG) reports using the International League Against Epilepsy classification (20). The subtyping of focal epilepsy was made by clinical history and ictal and interictal scalp EEG characteristics.

The attending physician determined the quantity of PER for each patient to prevent side effects and control seizures.

As habitual clinical practice, all the patients underwent a standardized neuropsychological evaluation in order to assess visuospatial skills [Rey–Osterrieth Complex Figure test (RCFT)] before adding PER (baseline; *n* = 46) and after 12 months of drug therapy (T1; *n* = 42).

At the same time, executive functions and non-verbal intelligence were evaluated through EpiTrack Junior test and standard progressive matrices (SPM).

The following factors were considered in our analysis: sex, age, age at onset of seizures, duration of epilepsy, seizure frequency, seizure types, seizure outcome, and concomitant PER and ASMs dose.

All parents kept a diary of seizures and AEs. The efficacy of PER treatment was measured considering the responder rate (at least 50% reduction in seizure frequency); tolerability was evaluated considering AEs reported by parents.

All participants were informed of the study objectives and methods, and the parents signed informed consent. The study complied with the rules of good clinical practice of the Declaration of Helsinki and approved by the local Ethics Committee.

### RCFT

RCFT is a neuropsychological assessment in which visuospatial perception and memory skills are assessed in both children and adults (21, 22).

The subjects were given a pencil and a blank sheet of paper. Subjects are initially asked to copy the figure (RCFT-Direct Copy) and after 3 min to reproduce it from memory (RCFT-Immediate Recall). There is no time limit for this test. The evaluation was based on 18 distinct elements of the figure, based on a two-point scale for a maximum of 36 points. When the graphic element is correct and positioned appropriately, two points are awarded; when the element is incomplete, incorrectly positioned, or distorted, one point is awarded; 0.5 points if incomplete and in the wrong place; 0 points when the item is missing or unrecognizable. Raw scores are converted to age-based percentile scores.

### EpiTrack Junior

EpiTrack Junior (23) is a test used to evaluate executive functions suitable for monitoring drug treatment as it is very sensitive. It consists of six tests (visual motor speed, inhibition, visual motor planning, mental flexibility, working memory, and mental fluency) from which an age-corrected total score is obtained.

The corrected total score is 49. An executive function deficit is given by a total score below 32; in particular, a score from 31 to 29 indicates a mild deficit, and a score ≤ 28 points indicates a significant deficit. A significant change in two successive measures is indicated by a loss of >2 points and a gain of >3 points.

### SPM

The Raven Progressive Matrices is a standardized tool used to assess non-verbal intelligence in individuals ranging from age 5 to adulthood (24). There are several versions; in our study, the SPM composed of five series of 12 elements of increasing complexity were used to analyze and encode visuospatial information.

Percentiles and age-weighted standard scores (mean = 100; standard deviation = 15) were derived from the raw scores. Scores ≥5th percentile or ≥70 standard are within the normal range (25).

### Statistical Analysis

All neuropsychological scores were reported as mean ± standard deviation (SD). The percentage of participants with a below normal score (<2 SD) was considered. The Kolmogorov–Smirnov test of normality was preliminarily performed to establish the distribution of the data. Given the presence of some data not normally distributed, non-parametric tests were used for the analyses.

Yates' corrected chi-square test was used for the comparison of proportions. The mean score comparison was performed using the Wilcoxon signed-rank test (paired samples) or the Mann–Whitney *U*-test (independent sample). The comparison of the mean scores in the independent samples was made with the Kruskal–Wallis *H*-test.

The relationship between the different variables was evaluated with the two-tailed Spearman rank correlation test. The correlations were explained as follows: <0.2, low; 0.21–0.40, fair; 0.41–0.60, moderate; 0.61–0.80, good; 0.81–1.00, very good.

Statistical Package for Social Science software, version 23.0 (26) was used to analyze the data. Our hypotheses were tested using Bonferroni adjusted alpha levels of 0.01 per test (0.05/5).

## Results

### Sample Characteristics

We recruited 46 adolescents aged between 12 and 18 years (mean age = 13.37 ± 1.51) affected by focal or generalized drug-resistant epilepsy, who started PER as a first or second ASM for a better seizure control. Two of them refused test administration at T1, and two were lost to follow-up.

All demographic and clinical features of the participants are summarized in Table 1; concomitant ASMs at baseline and their modification after 12 months are reported in Table 2. There were no significant differences between the main sample characteristics at baseline and T1.

**Table 1:** Demographic and clinical characteristics.

	**T0**	**T1**	**Statistics T1 vs. T0**
***N***	46	42	
**Sex**			χ^2^
Male	24 (52%)	22 (52%)	*p* = 0.846
**Age (years)**			Wilcoxon
Mean (SD)	13.37 (1.51)	13.39 (1.51)	*p* = 1.000
**Age at onset of epilepsy (years)**			Wilcoxon
M (SD)	7.02 (3.50)	7.07 (3.60)	*p* = 1.000
**Duration of epilepsy (years)**			Wilcoxon
M (SD)	6.59 (4.25)	6.60 (4.34)	*p* = 1.000
**Seizure type**			χ^2^
Focal–aware	10 (22%)	8 (19%)	*p* = 0.961
Focal–impaired awareness	22 (48%)	22 (52%)	*p* = 0.830
Generalized	14 (30%)	12 (29%)	*p* = 0.966
**Lobe**			χ^2^
Temporal	23 (72%)	21 (70%)	*p* = 0.906
Frontal	6 (19%)	6 (20%)	*p* = 0.843
Occipital	3 (9%)	3 (10%)	*p* = 0.728
**Side**			χ^2^
Left	19 (59%)	17 (57%)	*p* = 0.966
Right	13 (41%)	13 (43%)	*p* = 0.966
**Seizure frequency (per month)**			Wilcoxon
M (SD) Baseline	10.91 (6.38)	11.00 (6.91)	*p* = 1.000
M (SD) T1	–	5.52 (5.90)	
**Seizure outcome**			
Seizure reduction >50%	–	23 (55%)	
Seizure free		6 (14%)	
**Antiseizure drug load**			
M (SD) Baseline	1.63 (0.49)	1.64 (0.48)	
M (SD) T1	–	2.50 (0.66)	
**PER dose (mg/d)**			
M (SD)		3.40 (1.17)	
**Drop-out**	**Cause of PER discontinuation**	**PER dose (mg/d)**	
	Poor compliance	2	
	Poor compliance	3	
	Lost at follow-up	4	
	Lost at follow-up	2	

*M, mean; SD, standard deviation; T0, baseline; T1, 12 months follow-up; PER, perampanel*.

**Table 2:** Concomitant antiepileptic drug use at baseline and after 12 months follow-up.

**Concomitant ASM**	**Baseline (*N* = 46)**	**T1 (*N* = 42)**
Levetiracetam (LEV)	18 (39%)	16 (38%)
Valproid acid (VPA)	16 (35%)	14 (33%)
Carbamazepine (CBZ)	11 (24%)	6 (14%)
Ethosuximide (ETS)	8 (17%)	4 (10%)
Oxcarbazepine (OXC)	7 (15%)	5 (12%)
Lamotrigine (LTG)	6 (13%)	4 (10%)
Topiramate (TPM)	5 (11%)	2 (5%)
Clobazam (CLB)	5 (11%)	4 (10%)
Lacosamide (LCM)	3 (6%)	3 (7%)
Perampanel (PER)	0 (0%)	42 (100%)

*ASM, antiseizure medication; T1, 12 months follow-up*.

Seventy percent of patients had focal seizures (with or without impaired awareness); the remaining 30% had generalized seizures. PER was added as the first add-on in 17 patients (37%) and as the second add-on in the remaining ones. Patients received a variable dose of PER ranging from 2 to 8 mg (mean dose = 3.40 ± 1.17).

After 12 months of PER therapy, 23 patients (55%) had ≥50% seizure reduction, and six were seizure-free (14%). No significant AEs were reported with the exception of transient irritability (*n* = 3) and dizziness (*n* = 2), which did not require drug withdrawal.

### Baseline Assessment

On balance, analyzing the results obtained in RCFT-Direct Copy, a below normal score (<5th percentiles) was obtained by 13% (6/46) of the patients, and 15% (7/46) showed a score at the low limits of the norm (5th−15th percentiles). The RCFT-Direct Copy mean score of the total sample at baseline was 29.09 ± 4.91.

**Figure 1:**
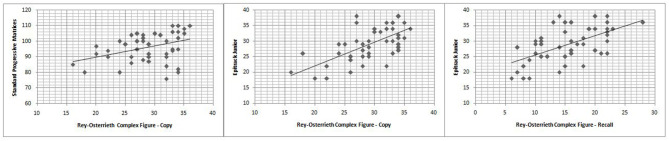
Correlation between Rey-Osterrieth complex figure test scores and Epitrack Junior or Standard progressive matrices scores.

The Spearman correlation test (Table 3) showed that RCFT-Direct Copy score was significantly related to executive functions (*r* = 0.614; *p* < 0.001) and non-verbal IQ (*r* = 0.419; *p* < 0.001), and there was no significant association with age, epilepsy duration, seizure frequency, age at onset of epilepsy, and ASM number (Table 2; Figure 1). There was also no significant difference in mean scores based on the following variables: sex [Mann–Whitney *U*-test (*n* = 46), *U* = 232.5, *p* = 0.478], seizure type [Mann–Whitney *U*-test (*n* = 46), *U* = 153.5, *p* = 0.091], side [Mann–Whitney *U*-test (*n* = 46), *U* = 96.5, *p* = 0.476], and lobe of seizure onset [Kruskall–Wallis *H*-test (*n* = 46), *H* = 2.511, *p* = 0.825].

**Table 3:** Spearman correlation analysis.

	**Rey-Osterrieth complex figure test (visual spatial skills)**	
	**Copy**	**Recall**
EpiTrack junior	***r*** **=** **0.614**	***r*** **=** **0.564**
(Executive functions)	***p*** **=** 0.000^*^	***p*** **=** 0.000^*^
SPM	***r*** **=** **0.419**	*r* = 0.211
(Non-verbal Intelligence)	***p*** **=** 0.004^*^	*p* = 0.160
Age	*r* = 0.215	*r* = 0.112
	*p* = 0.151	*p* = 0.460
Age at onset	*r* = 0.255	*r* = 0.192
	*p* = 0.087	*p* = 0.200
Epilepsy Duration	*r* = −0.236	*r* = −0.213
	*p* = 0.114	*p* = 0.155
Seizure frequency	*r* = −0.051	*r* = 0.142
	*p* = 0.736	*p* = 0.347
ASM number	*r* = −0.082	*r* = −0.129
	*p* = 0.589	*p* = 0.392
Perampanel dose	*r* = 0.039	*r* = 0.092
	*p* = 0.797	*p* = 0.542

SPM, standard progressive matrices; ASM, antiseizure medication. Asterisks (^*^) mark significant differences. Significant p value are in bold.

Analyzing RCFT-Immediate Recall, we found that 24% (11/46) of patients with epilepsy showed a score under the norm (<5° percentiles), whereas 21% (8/46) showed a score at the low limits of the norm (5–15°percentiles). The RCFT-Direct Copy mean score total at baseline was 15.52 ± 5.32. The Spearman correlation test (Table 3) showed that the RCFT-Immediate Recall scores were related to executive functions (*r* = 0.564; *p* < 0.001), and there was no significant association with non-verbal IQ, age, epilepsy duration, seizure frequency, age at onset of epilepsy, and ASM number (Table 2; Figure 1). There was also no significant difference in mean scores based on the following variables: sex [Mann–Whitney *U*-test (*n* = 46), *U* = 219.5, *p* = 0.326], seizure type [Mann–Whitney *U*-test (*n* = 46), *U* = 215.5, *p* = 0.839], side (Mann–Whitney *U*-test (*n* = 46), *U* = 113.5, *p* = 0.984], and lobe of seizure onset [Kruskall–Wallis *H*-test (*n* = 46), *H* = 0.243, *p* = 0.886].

### Comparison Between T1 and Baseline

After 12 months, a below normal RCFT-Direct Copy score was obtained by 16% (7/42) of patients, and a low-limit score of normal was obtained by 19% (8/42) of patients; on the RCFT-Immediate Recall test, 20% (9/42) of patients scored below normal (<5th percentiles), and 24% (10/42) scored at the low limits of normal (5th−15th percentiles).

**Figure 2:**
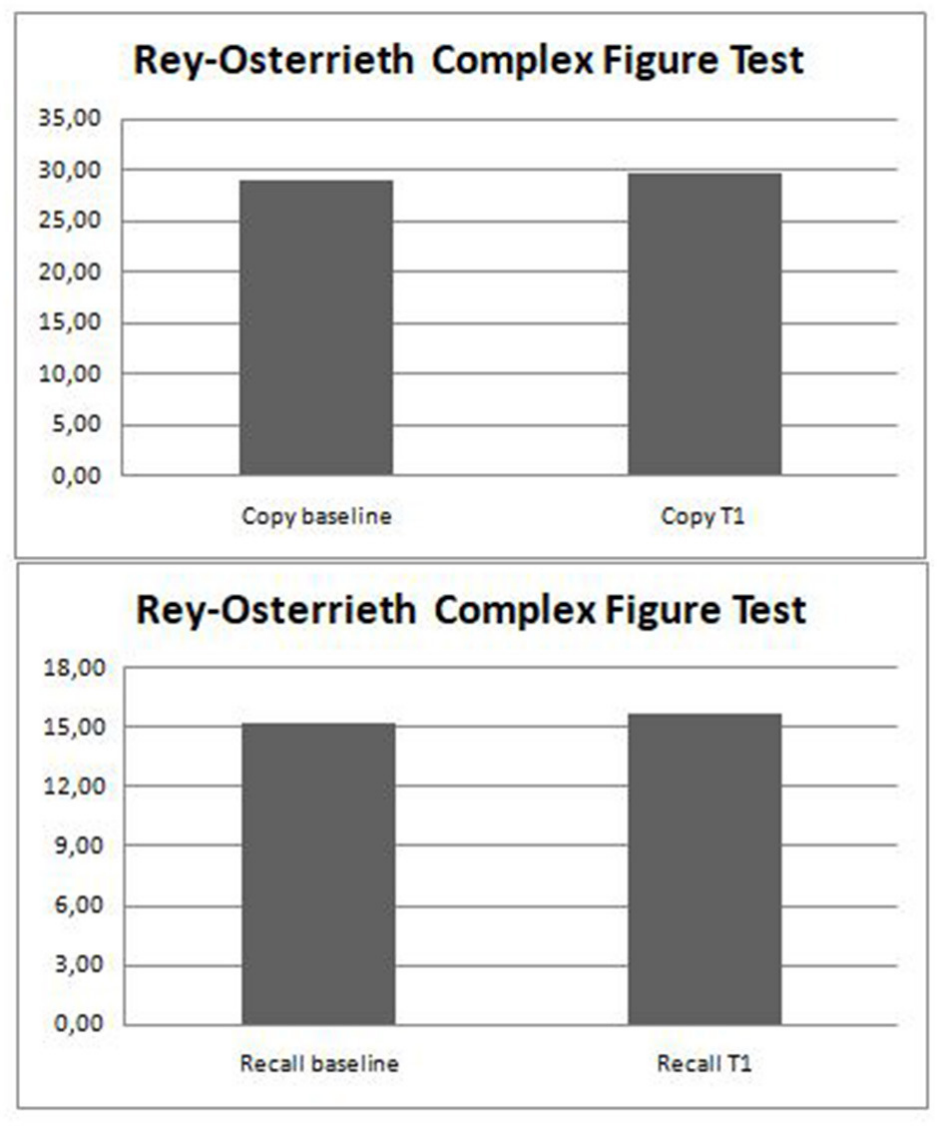
Changes in visuospatial skills after perampanel addition.

After 12 months, we discovered that the RCFT-Direct Copy and -Immediate Recall mean scores did not significantly differ from baseline (Table 4; Figure 2). All the results are summarized in Table 4.

**Table 4:** Baseline and outcome data following adjunctive treatment with Perampanel.

	**BASELINE**	**T1**	**Statistical analyses**
	***N* = 46**	***N* = 42**	**T1 vs. Baseline**
**RCFT (visual spatial skills)**			
**Copy**			Wilcoxon
M ± SD	29.09 ± 4.91	29.57 ± 3.76	Z = −0.897 0.370
**Recall**			Wilcoxon
M ± SD	15.52 ± 5.32	15.76 ± 5.43	Z = −1.023 0.306
**EPITRACK JUNIOR (executive functions)**			
M ± SD	28.98 ± 5.69		
**SPM (non-verbal Intelligence)**			
M ± SD	96.35 ± 9.38		

*RCFT, Rey-Osterrieth complex figure test; T1, 12 months follow-up; M, mean; SD, standard deviation; SPM, standard progressive matrices*.

## Discussion

With the insert of the new ASMs, which have similar effectiveness in the seizure control, a good tolerability on the cognitive profile is discriminating in the choice of the drug therapy (14). This aspect is particularly important in pediatric patients in which cognitive, executive, and visuospatial skills are indispensable for good school achievement and a good quality of life (27–29).

Our longitudinal observational study was based on 46 pediatric patients with focal or generalized drug-resistant epilepsy evaluated with neuropsychological tests for visuospatial perception and memory before and 12 months after the insert of PER as the first or second addition.

Initially, a score below normal was obtained by 13 and 24% of patients in visuospatial perception and memory, respectively, and 15 and 21% of patients scored at the lower limits of normal in these skills.

Our data confirm what has been already been described by previous research, which demonstrated a global impairment of visuospatial skills in epileptic patients compared with controls (30, 31). In general, visuospatial abilities appear to be more or less impaired even in children and adolescents with well-controlled seizures (32). Our study, in keeping with the study of Tallarita et al. (33), shows a greater impairment of visuospatial memory rather than visuospatial perception in patients with epilepsy.

The correlation analysis in our sample showed that visuospatial perception was significantly correlated with non-verbal intelligence, and visuospatial memory was significantly correlated with both non-verbal intelligence and executive functions (e.g., attention, memory of work).

Impairment of executive functions in children and adolescents with epilepsy has already been described in previous literature studies (34). Our results suggest that visuospatial memory deficit in adolescents with epilepsy could also be linked to impairment of executive functions; however, future research should be carried out to better investigate the extent and the nature of this correlation.

In our sample, visuospatial perception and memory were not significantly related to sex, age, epilepsy duration, age at onset of epilepsy, seizure frequency, side and lobe of seizure onset, and ASM number. The data from our study are in contrast to other research, which suggests that greater impairment of cognitive function may be caused by an earlier age of onset of seizures, which affect a developing neuronal system (35, 36). The relationship between impaired visuospatial memory capacity and the side/lobe of seizure onset is controversial (37). The study by Völkl-Kernstock et al. (38) showed significant impairment in visuospatial memory in children with benign childhood epilepsy with centro-temporal spikes compared with controls, which did not depend on the side of seizure onset and drug treatment. Finally, unlike other literature studies, in our study there was no relationship between the ASM number at baseline and visuospatial functions.

However, all these aspects should be further investigated on larger samples in order to increase statistical power.

In this study, at the 12-month reassessment, the visuospatial perception and memory skills were not significantly mutated from baseline, suggesting that PER does not have a negative impact on these functions.

Generally, the ASM number is negatively associated with cognitive performance although some drugs have a better tolerability profile than others (14).

The study of Meschede et al. (39) showed that also lacosamide and PER did not determine an impairment in general cognitive abilities. The subsequent study of Meador et al. (16) shows no difference in the PER group vs. the placebo group in global cognition skills, working memory, and attention.

Our previous experience also suggests that PER had a good tolerability profile in adolescents with epilepsy, showing that, after 12 months of add-on therapy with PER, there was no worsening of executive functions and emotional–behavioral profile (17).

Concerning specifically visuospatial skills, we recently conducted an observational study on 207 pediatric patients with different types of epilepsy well-controlled by antiseizure monotherapy (40). In this study, we assessed visuospatial memory and perception at time zero (before drug therapy) and after 12 months of monotherapy with valproic acid, levetiracetam, carbamazepine, ethosuximide, or oxcarbazepine. At reassessment, subjects taking valproic acid, ethosuximide, or carbamazepine performed significantly worse in visuospatial memory, and subjects taking levetiracetam and oxcarbazepine showed no significant changes.

In this perspective, our results are in line with our previous study, according to which newer ASMs, such as PER, levetiracetam, or oxcarbazepine have a better tolerability on the cognitive profile compared to the older ASMs, such as carbamazepine, valproic acid, and ethosuximide.

In the present study, PER was effective for seizure reduction with a response rate of >50% in 23/42 subjects (55%), and 6/42 subjects are seizure-free (14%) after 12 months of therapy. No significant side effects were reported.

Other studies controlled against placebo/other ASMs and with larger sample sizes are needed to confirm our results.

## Conclusions

In conclusion, PER was well-tolerated in adolescents with focal and generalized seizures. Visuospatial memory and perception were not significantly affected by PER therapy. These results, therefore, suggest that PER has a good tolerability contour in adolescence even in the medium/long term.

## Data Availability Statement

The raw data supporting the conclusions of this article will be made available by the authors, without undue reservation.

## Ethics Statement

The studies involving human participants were reviewed and approved by Comitato Etico Campania Sud. Written informed consent to participate in this study was provided by the participants' legal guardian/next of kin.

## Author Contributions

FO conceptualized the work. GP analyzed the data and drafted the manuscript. CP, VV, and CS performed psychometric measurements and analyzed the data. GQ researched the data literature. MR supervised the work. All authors have agreed to this final version and all authors participated in a meaningful way in the preparation of the manuscript.

## Conflict of Interest

FO and GP participated at advisory boards and scientific meetings for Eisai. The remaining authors declare that the research was conducted in the absence of any commercial or financial relationships that could be construed as a potential conflict of interest.
